# Supplementary material for: Prevalence and Characterization of Quinolone-Resistance Determinants in Escherichia coli Isolated from Food-Producing Animals and Animal-Derived Food in the Philippines
Source: Antibiotics (Basel). 2021 Apr 9;10(4):413. doi: 10.3390/antibiotics10040413 (PMC8068814; doi:10.3390/antibiotics10040413)
Supplement: Supplementary file 1 [file antibiotics-10-00413-s001.pdf]

Supplementary Table. Antimicrobial susceptibility of *E. coli* (n = 791) isolates from food animals and food products.

Antimicrobial agents	*E. coli*, n (%)		
	Susceptible	Intermediate	Resistant
Tetracycline	300 (37.9)	36 (4.6)	455 (57.5)
Ampicillin	313 (39.6)	30 (3.8)	448 (56.6)
Sulfamethoxazole-trimethoprim	442 (55.9)	3 (0.4)	346 (43.7)
Chloramphenicol	486 (61.4)	21 (2.7)	284 (35.9)
Streptomycin	397 (50.2)	157 (19.8)	237 (30.0)
Nalidixic acid	497 (62.8)	94 (11.9)	200 (25.3)
Kanamycin	582 (73.6)	93 (11.8)	116 (14.7)
Ciprofloxacin	642 (81.2)	57 (7.2)	92 (11.6)
Colistin	712 (90.0)	1 (0.1)	78 (9.9)
Amoxicillin-clavulanic acid*	308 (85.3)	21 (5.8)	32 (8.9)
Imipenem	669 (84.6)	52 (6.6)	70 (8.8)
Gentamicin	707 (89.4)	17 (2.1)	67 (8.5)
Cefoxitin	686 (86.7)	39 (4.9)	66 (8.3)
Cefotaxime*	307 (85.0)	34 (9.4)	20 (5.5)
Ceftazidime	730 (92.3)	37 (4.7)	24 (3.0)
Cefepime	773 (97.7)	0 (0.0)	18 (2.3)
Meropenem*	349 (96.7)	9 (2.5)	3 (0.8)

*Only 121 samples were tested to Amoxicillin-clavulanic acid, Cefotaxime, Meropenem
